# Caveolin-3 promotes glycometabolism, growth and proliferation in muscle cells

**DOI:** 10.1371/journal.pone.0189004

**Published:** 2017-12-05

**Authors:** Lina Shang, Tingting Chen, Yufeng Deng, Yiyuan Huang, Yuanheng Huang, Jing Xian, Wensheng Lu, Lihui Yang, Qin Huang

**Affiliations:** 1 Department of Physiology, School of Basic Medical Sciences, Guangxi Medical University, Nanning, Guangxi, China; 2 Department of Endocrinology, The First Affiliated Hospital of Guangxi Medical University, Nanning, Guangxi, China; 3 Department of Endocrinology, The People’s Hospital of Guangxi Zhuang Autonomous Region, Nanning, Guangxi, China; University of Cincinnati College of Medicine, UNITED STATES

## Abstract

**Objective:**

Caveolin-3 (CAV3) protein is known to be expressed specifically in various myocytes, but its physiological function remains unclear. CAV3, located at the cell membrane, may promote the sensitivity of the Akt signaling pathway, which is closely related to glucose metabolism and to cell growth and proliferation.

**Methods:**

The CAV3 gene was stably transfected into C2C12 muscle cells, and the effects were evaluated by biochemical assays, WB and confocal microscopy for the observation of cellular glucose metabolism, growth and proliferation, and the effect of CAV3 on the Akt signaling pathway with no insulin stimulation.

**Results:**

After C2C12 cells were transfected with the mouse CAV3 gene, which increased CAV3 expression, the abundance of the CAV3 and GLUT4 proteins on the cell membrane increased, but the total GLUT4 protein content of the cell was unchanged. Glucose uptake was increased, and this did not affect the glycogen synthesis, but the cell surface area and cell proliferation increased. While there were significant increases in p-Akt and p-p70s6K, which is a downstream component of Akt signaling, the level of GSK3β protein, another component of Akt signaling did not change.

**Conclusions:**

The muscle, CAV3 protein can activate Akt signaling, increase GLUT4 protein localization in the cell membrane, increase glucose uptake, and promote myocyte growth and proliferation. CAV3 protein has a physiological role in glycometabolism, growth and proliferation, independent of insulin stimulation.

## Introduction

Caveolin (CAV) is a Caveolae-associated protein in cell membranes. The Caveolin gene family has three subtypes: CAV1, CAV2 and CAV3. CAV3 protein was first cloned and identified in 1996 and is specifically expressed in muscle cells, including skeletal muscle, cardiac muscle and smooth muscle cells, and is therefore also known as M-caveolin. The Caveolin-3 gene is located on human chromosome 3 and produces a protein consisting of 151 amino acids. It consists of an N-terminal region, transmembrane region and C-terminal region. Its N-terminal scaffolding domain (CSD) regulates a variety of signaling molecules including eNOS, G-protein, adrenergic receptor, protein kinase C monomers, and Src family protein kinases, and it has substantial effects on numerous aspects of muscle physiology, including muscular dystrophin, cholesterol transport, intracellular signaling, tumor suppression, and myocyte synthesis [1], but its physiological function in skeletal muscle is not yet fully understood.

Previous research showed that CAV3 proteins become increasingly abundant during the development of muscle cells and that they are involved in the formation of cell myotubes and differentiation [2, 3], the promotion of insulin receptor (IR) sensitivity, and the activation of the PI3K/Akt signaling pathway. Lack of CAV3 caused cell immaturity, muscle atrophy and increased blood glucose [4, 5]. The abovementioned research indicates that CAV3 is required for the growth and maturation of muscle cells, but the details require further exploration. Our previous study determined that CAV3-P104L mutations lead to impaired glucose metabolism. In this study, we observed the precise effect of increased CAV3 protein on cell morphology, growth, proliferation and glucose metabolism, and we explored the physiological function of CAV3.

## Materials and methods

### Cell culture and transfection

The mouse skeletal muscle cell line C2C12 (Shanghai Institutes for Biological Sciences, China) was maintained in a proliferation medium, DMEM (Gibco, 25 mM D-Glucose) containing 10% FBS (Gibco, Invitrogen), streptomycin (100 μl/ml) and penicillin (100 μl/ml) under conventional culture conditions: 5% CO2 and 37°C in a humidified incubator. Cells were approximately 70% confluent at 3 to 4 hours before transfection. Based on Invitrogen’s recommended DNA plasmid concentration of 0.5 to 5 μg/μL, Lipofectamine 3000 was used to transfected C2C12 cells with empty vector + eGFP (NC) or with wild type CAV3 + eGFP (WT). The expression vector was constructed by the Guangzhou GeneCopoeia Company (USA). 24 hours after transfection, G418 was added to the cultured cells for the selection of positive clones to construct two stable cell lines, which were then screened by fluorescence inversion microscopy.

### Western blot analysis and antibody

Total protein was extracted from cultured C2C12 cells. Cells were rinsed twice with PBS at 4°C and subsequently harvested in cold lysis buffer (150 mM NaCl, 1% Triton X-100, 1% sodium deoxycholate, 0.1% SDS, sodium orthovanadate, sodium fluoride, EDTA, leupeptin, and a mix of protease inhibitors). Samples were scraped with cell curettes, and subsequently, the cells were shaked with an oscillator and centrifuged at 12,000 rpm for 30 min at 4°C. The protein concentration was obtained using the BCA kit. Electrophoresis was performed using 5% concentration gels and 10–12% separation gels to facilitate the visualization of the different molecular weights. Samples of approximately 20–50 μg were added to the loading buffer. After 5 min of boiling, the cell lysates were separated by 10% sodium dodecyl sulfate polyacrylamide gel electrophoresis (SDS-PAGE) and transferred to a polyvinylidene fluoride membrane (Millipore) in Tris–glycine buffer containing 20% methanol. The membranes were washed with TBST (50 mg Tris-HCL, pH 7.6, 150 mM NaCl, 0.2% Tween 20), blocked with 5% skim milk for 1 h and incubated overnight with primary antibodies: Akt (2920, 1:1000 dilution, Cell Signaling Technology), p-Akt^ser473^ (12694, 1:1000 dilution, Cell Signaling Technology), p-AMPKα^Thr172^ (2535, 1:1000 dilution, Cell Signaling Technology), AMPKα (2532, 1:1000 dilution, Cell Signaling Technology), p-GSK3β (9323, 1:1000 dilution, Cell Signaling Technology), GSK3β (9315, 1:1000 dilution, Cell Signaling Technology), p-p70s6K^Thr389^ (9205, 1:1000 dilution, Cell Signaling Technology), p70s6K (2708, 1:1000 dilution, Cell Signaling Technology), GFP (J20625, 1:500 dilution, TransGen), GAPDH (YM1038, 1:500 dilution, ImmunoWay), CAV3 (sc-5310, 1:100 dilution, Santa Cruz), or GLUT4 (sc-01608, 1:100 dilution, Santa Cruz). After 5 washes with TBST, membranes were incubated for 2 h at room temperature with the IRDye800CW coupling secondary antibody. Next, the membranes were washed 3 times and visualized using a Li-Cor Odyssey infrared imager (Li-Cor Biosciences, Lincoln, NE). Integrated intensities of the 800-nm infrared signal for each band were calculated using the software associated with the Li-Cor Odyssey infrared imaging system.

### Confocal microscopy

Stably transfected cells cultured on 6-well plates were washed 3 times with PBS and fixed for 30 min at room temperature with 4% paraformaldehyde. Subsequently, fixed cells were rinsed with PBS, incubated with 10% donkey serum at room temperature for 30 min, and then immediately incubated overnight at 4°C in the presence of the diluted anti-CAV3 and anti-GLUT4 antibodies (1:250, Santa Cruz Biotechnology). Next, cells were washed with PBS 3 times in the dark and incubated with Alexa Fluor 594 (1608643, 1:1000, Invitrogen) and Alexa Fluor 647 (1692912, 1:1000, Invitrogen), for evaluation using 2 different excitation wavelengths at 37°C for 2h. The cells were observed using a laser confocal microscope (Nikon).

### Cell surface area measurements

To assess whether the transfection of the exogenous CAV3 gene promoted cell growth, we measured the cell surface area in the NC and WT groups by using a confocal microscope and Image-Pro Plus software. Cells were seeded at a density of 1 × 10^4^ cells/mL on a 6-well plate and cultured for 48 h in DMEM with G418. After 48 hours, the cells were observed under the confocal microscope. 3 visual fields were randomly selected for each well, and a total of 9 fields were collected for each group. Cell surface area was analyzed using Image-Pro Plus (Media)[6], which enables the user to select cells within an image for border detection and for calculation of their surface areas; these areas are determined by calculating the sum of the pixels within the boundary of each myocyte.

### Cell growth curve

Two groups of cells were seeded in 24-well plates at the same density. 5 wells were prepared for each group. The cells were counted after 24 h and every 24 h there after for 4 days, and medium was changed every 48 h. Based on these results, the C2C12 cell growth curve was constructed.

Cell proliferation assays were performed using the cell counting kit-8 (CCK-8) (Dojindo, Kumamoto, Japan) according to the manufacturer’s instructions. Two groups of cells were seeded into 96-well plates at a density of 1 × 10^3^ cells/100 μL/well and treated with CCK-8 for 5 days. Cell growth was visualized with SigmaPlot 12.5. 5 wells were prepared for each group, and the experiment was repeated 3 times.

### Determination of glucose uptake and glycogen synthesis

Stably transfected cells were seeded at a density of 1 × 10^6^ cells per 60 mm dish. 5 dishes were established for each group. After 48 hours, the supernatant was collected and subjected to the glucose oxidase/hydrogen peroxide (GOD/POD) assay according to the manufacturer’s instructions for the Glucose Assay kit (Nanjing Jiancheng Bioengineering Institute, China)[7]. The amount of glucose remaining in the culture wells of each group was subtracted from the average content of glucose in the culture medium without cells representing the glucose consumption, which reflected the glucose uptake. After the supernatant was removed, cells were digested with trypsin, and the total amount of glycogen was measured by using the Glycogen Assay kit (Nanjing Institute of Bioengineering, Nanjing, China)[8, 9].

### Statistical analysis

Data are expressed as the mean ± standard deviation (`x ± s). Statistical significance was determined using Student’s t-test in SPSS20.0 software. The difference between the two groups was considered to be significant when P <0.05.

## Results

### Transfection of CAV3 increased the surface area and the proliferation rate in C2C12a cells

**Fig 1:**
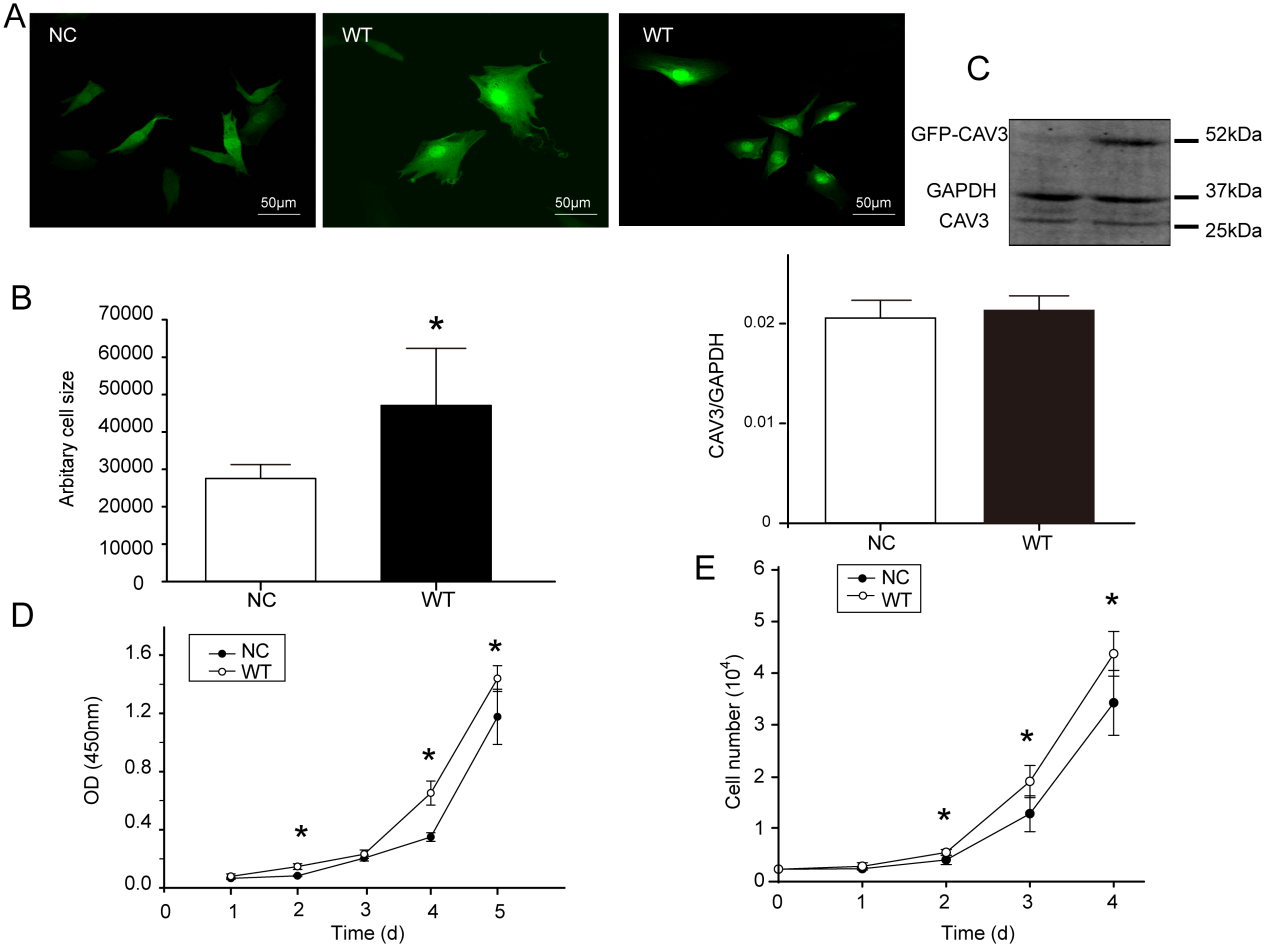
Effects of CAV3 transfection on cell surface area and cell numbers. (A) Two groups of transfected cells viewed using confocal microscopy; scale bar, 20 μm. (B) The cell surface areas were analyzed using Image-Pro Plus, and the relative cell sizes are expressed as the mean ± SD (magnification: 40×). Data are from three fields per group and from three separate experiments, *p<0.05 compared with the NC group. (C) CAV3 endogenous expression: C2C12 cells normally contains a certain amount of CAV3. Endogenous CAV3 protein is approximately 25 kDa, whereas GFP-CAV3 recombinant protein is approximately 52 kDa. Therefore, a band at 25 kDa was detected in both the NC and WT groups, as expected, showed the endogenous CAV3 protein. In contrast, only the WT group showed the recombinant protein. The bar chart depicts expression of the endogenous protein. Data are presented as the mean ± SD. n = 5, *p< 0.05. (D) The cell growth curves determined using CCK-8 kit for 5 days. (E) Cell growth curves over 4 days. The growth curves of cells were determined by counting cells in a 24-well plate with standard medium and conditioned medium (n = 5).

As shown in Fig 1C, for detection of recombinant GFP-CAV3 protein, only GFP was detected in the NC group, whereas the GFP recombinant protein was detected in the WT CAV3 group, indicating the successful expression of the CAV3 vector in the C2C12 cells. There was no significant difference in the endogenous expression of CAV3 between the two groups. Compared with the NC group, the surface area of the C2C12 cells in the WT group increased significantly (P = 0.002) (Fig 1A and 1B). The growth curve shows that the cell numbers in both groups increased over time, but the growth rate of the WT group was significantly higher than that of the NC group (Fig 1D and 1E) S1 File.

### Transfection of CAV3 increased the shift of GLUT4 to the cell membrane

**Fig 2:**
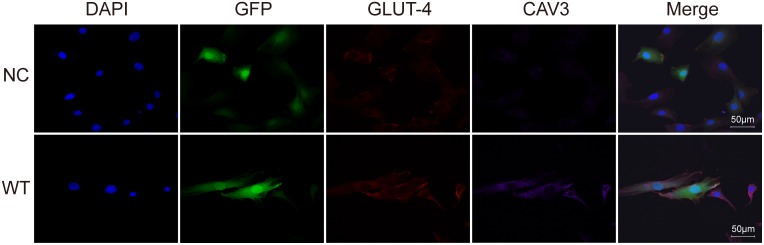
Increased expression of CAV3 promoted a shift of GLUT4. The expression of CAV3 and GLUT4 was detected by confocal microscopy (magnification: 40×). Representative photomicrographs show C2C12 cells transfected with an empty vector and with WT-CAV3, followed by double staining to detect CAV3 (purple) and GLUT4 (red). Nuclei ere stained blue with DAPI. Cells were transfected with eGFP (green). Transfection of CAV3 increased the level of CAV3 protein and promoted a shift of GLUT4 to the cell membrane. Scale bars = 50 μm.

As observed by confocal microscopy, CAV3 protein was mainly localized in the cytoplasm and the cell membrane (Fig 2), and the expression of total CAV3 in the WT group was higher than in the NC group. In the NC group, GLUT4 protein was mainly distributed in the cell membrane and around the nucleus, but with the increase in CAV3 in the WT group, most of the GLUT4 was located in the cell membrane.

### Transfection of CAV3 promoted glucose uptake but did not impact total glycogen in cells

**Fig 3:**
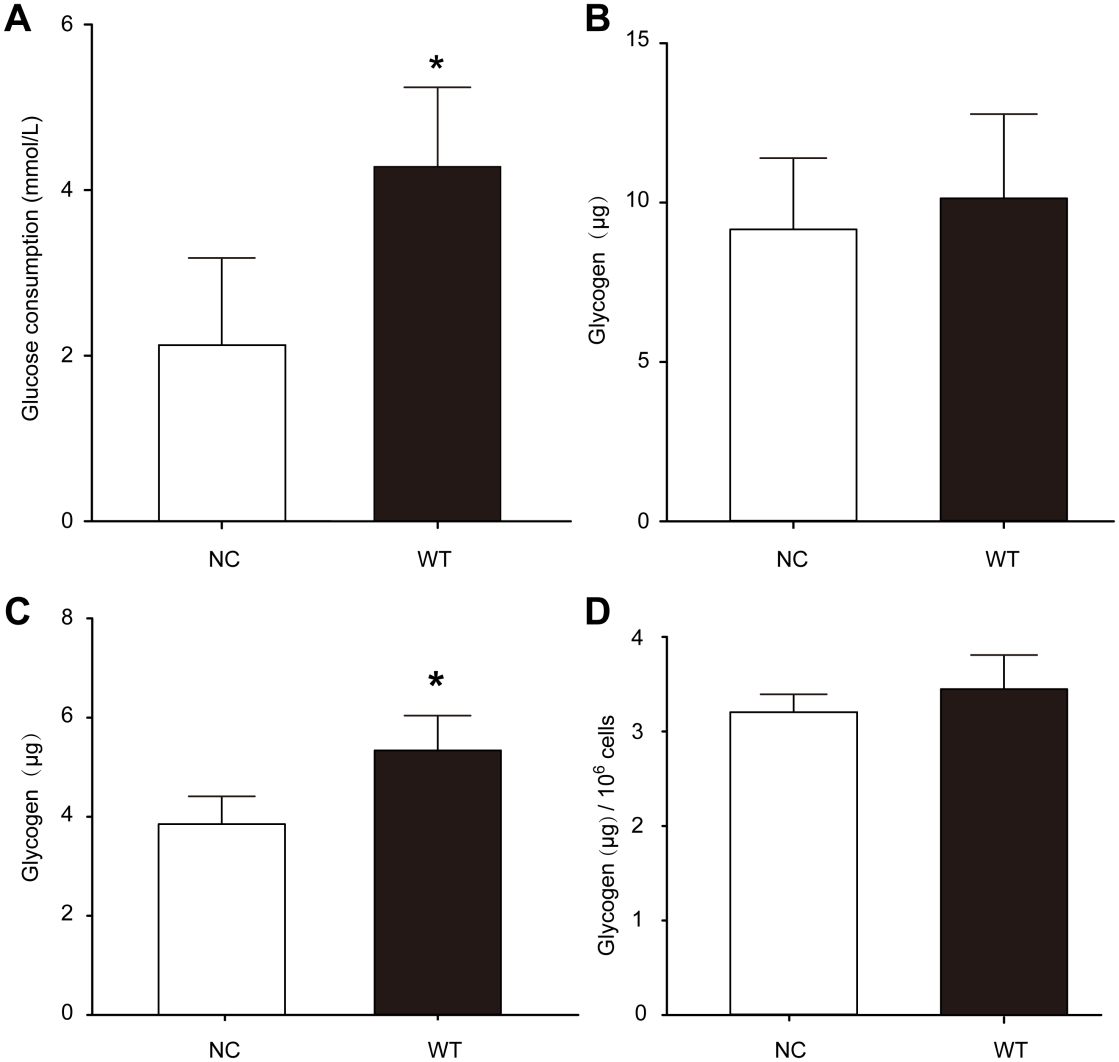
Transfection of CAV3 promoted glucose consumption but not glycogen synthesis. (A) Compared with the NC group, glucose consumption was significantly increased in the WT group after CAV3 transfection. (B) Compared with NC the group, glycogen was not increased when balanced with unit cell numbers in the WT group. (A, B) Cells were cultured for 48 h, * p<0.05, n = 5. (C) The total amount of glycogen synthesis was increased, (D) but glycogen synthesis of the unit of cells according to proliferation was not obviously increased. Unit cell glycogen content: after the cell count, considering 1 × 10^4^ cells as a unit, the glycogen content of each unit was calculated. (C, D) Cells were cultured for 6 days, * p<0.05, n = 5.

Our results showed that glucose consumption (Fig 3A) in the WT group was increased compared with the NC group (p = 0.006), indicating that CAV3 promotes glucose uptake into cells. However, there was no significant difference in the total amount of glycogen between the WT group (Fig 3B) and the NC group (P = 0.454). When the cells were cultured for six days, the total amount of glycogen synthesis was increased (Fig 3C), but glycogen synthesis of the unit of cells according to proliferation was not obviously increased (Fig 3D),. indicating that altered CAV3 expression is not sufficient to change the glycogen levels in the cells S2 File.

### Transfection of CAV3 increased p-Akt, p-AMPK and p-p70s6K but did not affect the total protein of GLUT4 and GSK3β

**Fig 4:**
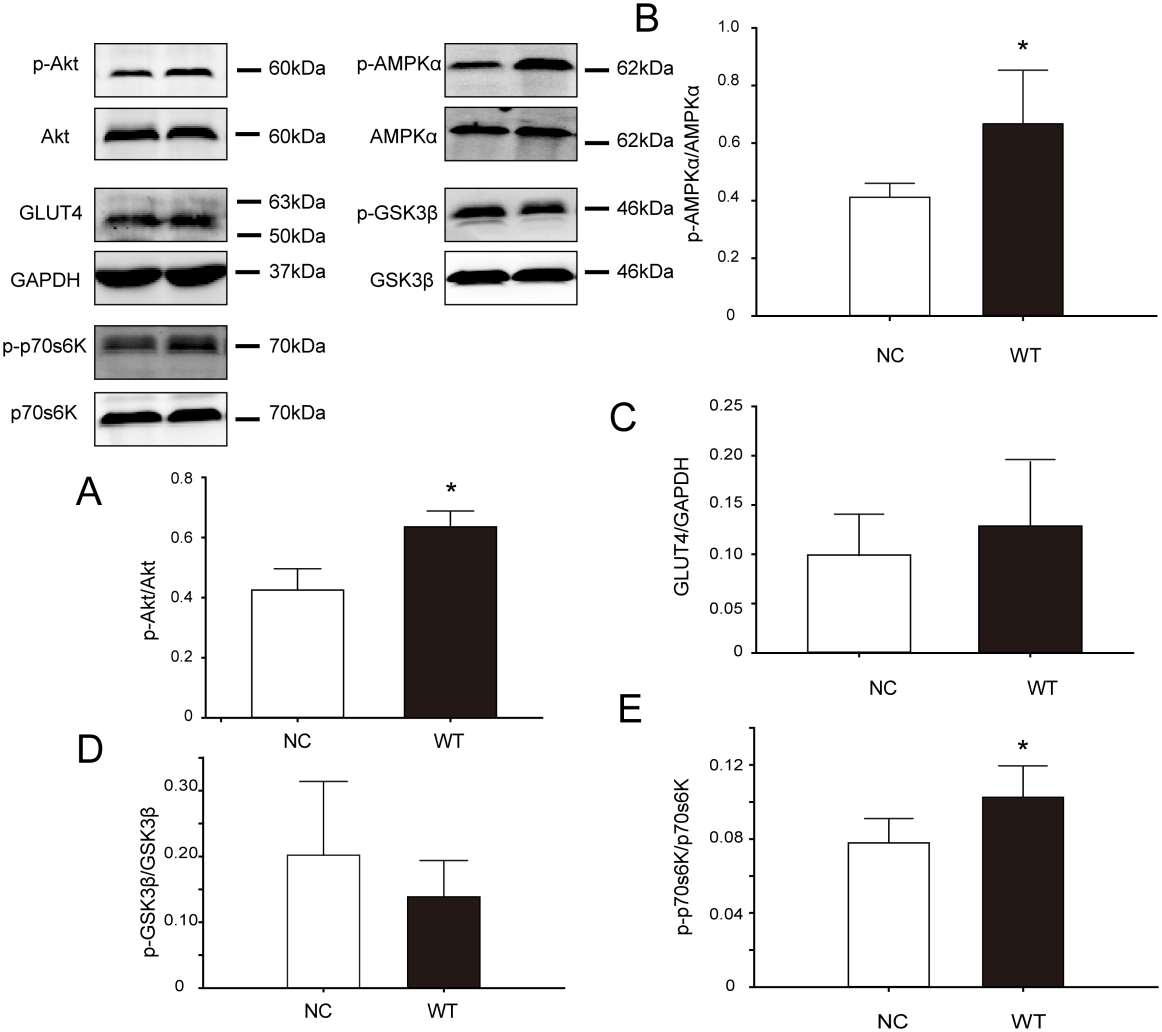
Changes in the expression of key signaling proteins in C2C12 cells after CAV3 transfection. Total proteins from NC and WT cells were separated by SDS-PAGE and measured via western blot analysis. Representative immunoblots for the indicated proteins, with GAPDH as a loading control, are shown. The bar plots show semi-quantitative densitometry analysis for the normalized expression of the indicated protein. The p-Akt/Akt, p-AMPK/AMPK and p-p70s6K/p70s6K ratios were significantly increased in the WT group. Data are presented as the mean ± SD, n = 5, * p<0.05.

Akt is a key factor in the PI3K/Akt signaling pathway, and Akt activation requires phosphorylation. Western blot analysis showed that p-Akt expression was significantly higher in the WT group than in the NC group (P = 0.010 Fig 4A), whereas there was no difference in total Akt expression between the two groups. Three downstream molecules in the Akt pathway were also analyzed: there was no change in the glucose transporter GLUT4 when comparing the WT group with the NC group (Fig 4D); the protein synthesis regulatory factor p70s6K was activated, given that its phosphorylation had increased in the WT group compared with the NC group (Fig 4E); and the glycogen synthase kinase GSK3β (Fig 4C), which may be negatively correlated with glycogen synthesis, showed no change in the WT group compared with the NC group. Moreover, AMPK is also reported to regulate GLUT4 translocation[10]. Western blot results showed that AMPK is activated in the WT group (P = 0.018 Fig 4B) S3 File.

## Discussion

Previous reports on the physiological function of CAV3 in muscle cells in general are primarily concentrated in four areas: 1) CAV3 may bind to IRS-1, enhancing the sensitivity of insulin signaling and GLUT4 translocation, suggesting that CAV3 promotes glucose uptake mediated by the insulin receptor (IR) signaling pathway in muscle cells [5, 11–14] and may also activate Akt, which leads to increased protein synthesis and cell diameter [15, 16]. 2) CAV3 could maintain the K+ channel, the Ca2+ channel and the Na+-K+ -ATP enzyme that are closely related to cell survival and the contraction function. 3) Morphological observations suggest that CAV3 is necessary for the formation of T structures, for cell differentiation and for maturation [3, 4]. 4) CAV3 may be involved in maintaining the stability of the cell membrane [17]. In this study, we report several observations on a stably transfected cell line overexpressing CAV3 without insulin stimulation.

### Increased expression of CAV3 promoted the growth and proliferation of muscle cells

In the presence of high level of glucose, after CAV3 proteins expression increased, the cell proliferation rate increased. CAV3 protein also caused the cell surface area and diameter to increase, as observed by confocal microscopy. A previous report showed increased cell diameter after transient transfection of the CAV3 gene into C2C12 cells [16]. These data directly show that CAV3 not only promotes the growth of muscle cells but also accelerates their division and proliferation.

### Increased expression of CAV3 promoted glucose uptake but did not change glycogen levels in cells

In mammals, skeletal muscle accounts for 40% of the total body weight and more than 50% of the total energy consumption. Under normal conditions, skeletal muscle can use the glucose in circulation and the glycogen in storage to maintain normal metabolism and function. The muscles use approximately 95% of systemic glucose under high-glucose and high-insulin-stimulation conditions [18].

Our previous study found that when stimulated with high level of insulin, up-regulating CAV3 protein expression in C2C12 cells by transfection of a mutated gene led to reduced glucose uptake and glycogen content [5]. Another report showed that mice deficient in CAV3 protein developed insulin resistance and impaired insulin signaling pathways in skeletal muscle, with no effect on hepatic insulin signaling, resulting in decreased glucose uptake in skeletal muscle and increased blood glucose levels [19].

Our results showed that following an increase in CAV3 protein, glucose uptake approximately doubled, and total glycogen content increased gradually in cells, but the glycogen content did not markedly increase after eliminating the factor of cell proliferation by using calculations normalized to the number of cells, suggesting that CAV3 protein only promotes glucose uptake in the muscle cells and does not affect total cell glycogen. Beyond an increase in the CAV3 protein, other factors, such as insulin stimulation or contraction stimulation, may be required to support cellular glycogen synthesis. It has been reported that GSK3β inhibited cell proliferation in other kinds of cells [20]. For the C2C12 cell line with increased CAV3, faster cell growth and proliferation require the consumption of more energy and materials from intracellular glucose, so there may not be enough glucose present to increase glycogen synthesis. Here, we also speculate that cell growth, proliferation and glycogen synthesis, all of which are related to glucose consumption, may modulate and influence each other.

### Increased expression of CAV3 activated the Akt signaling pathway and its downstream effectors GLUT4 and p-p70s6K but did not affect GSK3β

The IR signaling pathway is known to mediate cell glucose, lipid and protein metabolism, including promoting glucose transport, utilizing glycogen and promoting glycogen synthesis, inhibiting gluconeogenesis and glycogenolysis, promoting protein synthesis and inhibiting protein decomposition. In CAV3 knockout mice, the IR protein in skeletal muscle decreased rapidly after 15 min of insulin stimulation, which led to the occurrence of insulin resistance, decreased glucose uptake and decreased glycogen synthesis [21]. Muscular injections of CAV3 protein can cause IR signal restoration in muscle tissue [19]. Overexpression of CAV3 enhanced the tyrosine phosphorylation of IR substrate 1 (IRS-1), which can activate the PI3K/Akt signaling pathway. Akt is a key factor that participates in multiple signaling pathways involved in cell survival, growth and metabolism, and it is related to the downstream substrates GLUT4, GSK3β and p70s6K [15, 16, 22, 23].

Our results showed that without insulin stimulation and with increased CAV3 expression, the phosphorylation of Akt increased by approximately 47.74%, and membrane location of GLUT4 increased (at least in part from the PI3K/Akt signaling pathway), but GSK3β, which is related to glycogen synthesis, was not affected. This finding is consistent with unchanged cellular glycogen levels. Additionally, the signaling molecule p70s6K, which is related to protein synthesis was also affected. We think that increased expression of CAV3 could activate Akt, which in turn activates p70s6K, thereby increasing protein synthesis and enlarging the cells.

### Increased expression of CAV3 promoted the non-insulin signaling pathway

The glucose uptake of tissue can be either insulin dependent or non-insulin dependent[24, 25]. Under postprandial hyperglycemia, insulin-stimulated glucose uptake by skeletal muscle increases; non-insulin- mediated glucose uptake also increases in skeletal muscle but not in the central nervous system [18]. We did not use insulin, and in C2C12 myoblasts cultured in DMEM with high glucose, p-Akt was significantly increased, by approximately 50%; the amount of GLUT4 protein located in the cell membrane increased; glucose uptake and protein synthesis increased; and the cell growth and proliferation rates increased. However, we did not find any obvious changes in GSK3β, which is related to glycogen synthesis indicating that cellular effects through the insulin signaling pathway did not play a role, or at least that most of the regulation by CAV3 followed non-insulin-mediated signaling pathways. In other words, glycogen synthesis canbe induced by insulin signaling downstream of increased CAV3 but not by insulin-independent signaling downstream of increased CAV3, which promotes glucose uptake in muscle cells. So CAV3 may promote both the insulin and non-insulin signaling pathways such that the special physiological functions in muscle cells are implemented. Glycogen synthesis was not increased, with the rapid growth and proliferation of C2C12 cells, glycogen consumption might increase.

GLUT4 is the most important transporter of glucose in skeletal muscle cells. Stimulated by insulin, GLUT4 translocates to the cell membrane, where it promotes cellular glucose intake and eventually reduces blood glucose [11, 14, 26, 27]. As observed using confocal microscope, increased CAV3 in muscle cells lead to increased GLUT4 localization in the cell membrane despite not affecting the total GLUT4 protein in cells, so the glucose uptake of muscle cells was accelerated. AMPK which regulates glucose metabolism is a non-insulin-activated signaling molecule, and it transfers the GLUT4 to the membrane [24]. It is the main mechanism by which GLUT4 is translocated to the plasma membrane due to exercise, hypoxia and other factors [28]. We examined total and phosphorylated AMPK, and we found that AMPK was activated, further confirming our previous confocal results: CAV3 increased GLUT4 localization at the cell membrane. And Rab GTPases had be reported to affect translocation of GLUT4 to the cell membrane, so we will include it in our further exploration [29].

In summary, in C2C12 muscle cells cultured in normal high-glucose DMEM, transfection and overexpression of CAV3 increased the phosphorylation of Akt, AMPK and p70s6K, increased the plasma membrane localization of GLUT4 protein, and increased glucose uptake, cell growth and proliferation. We demonstrated that the increased CAV3 protein promoted growth and glucose uptake, indicating that CAV3 plays an important physiological role in skeletal muscle cells.

This study explored the precise physiological function of CAV3 in muscle cells. For patients with myopathy with muscle weakness and atrophy, CAV3 gene transfection may help to improve muscle metabolism and increase the number of muscle cells, thereby restoring the morphology and function of muscle fibers. Muscle CAV3 may activate the insulin and non insulin pathways independently, leading to a variety of physiological effects.

## Supporting information

S1 File Relevant data underlying the findings described in manuscript. (PDF)

S2 File Relevant data underlying the findings described in manuscript. (PDF)

S3 File Relevant data underlying the findings described in manuscript. (PDF)
