# Correction: Caveolin-3 promotes glycometabolism, growth and proliferation in muscle cells

**DOI:** 10.1371/journal.pone.0197190

**Published:** 2018-05-07

**Authors:** The PLOS ONE Staff

In the Funding section, the grant number from the funder National Natural Science Foundation of China is listed incorrectly. The correct grant number is: 81660360. The publisher apologizes for the error.
