# Supplementary material for: Caveolin-3 promotes glycometabolism, growth and proliferation in muscle cells
Source: PLoS One. 2017 Dec 5;12(12):e0189004. doi: 10.1371/journal.pone.0189004 (PMC5716543; doi:10.1371/journal.pone.0189004)
Supplement: S1 File — (PDF) [file pone.0189004.s001.pdf]

Arbitrary cell size

NC	WT
31855	65262
31133	43875
33252	40019
26873	36132
29553	32725
26506	34808
26264	34960
23465	43929
22814	73451
23805	66439

Cell number(10^4)

Group	1 day	2 day	3 day	4 day
NC	0.2	0.5	1.875	3.125
NC	0.2	0.375	1.125	3.75
NC	0.2	0.375	1.25	4.125
NC	0.25	0.25	1.125	3.625
NC	0.2	0.4	1	2.5
WT	0.25	0.625	2.375	3.75
WT	0.25	0.5	1.625	4.75
WT	0.375	0.5	2	4.75
WT	0.2	0.5	1.875	4.5
WT	0.2	0.5	1.625	4.125

EndogenousCAV3/GAPDH

	NC	WT
	0.0185	0.0205
	0.0232	0.0233
	0.0195	0.0198
	0.0211	0.0223
	0.0205	0.021
$\bar{x}$	0.0206	0.02138
s	0.0018	0.00141

CCK-8

Group	1 day	2 day	3 day	4 day	5 day
NC	0.0678	0.0852	0.2234	0.3206	1.0166
NC	0.0768	0.0782	0.1704	0.3216	1.1176
NC	0.0568	0.0852	0.2154	0.3656	1.4966
NC	0.0618	0.0762	0.2064	0.3906	1.0646
NC	0.0638	0.0892	0.2094	0.3496	1.1836
WT	0.1078	0.1162	0.2224	0.6716	1.4836
WT	0.0738	0.1572	0.2394	0.6056	1.5316
WT	0.0698	0.1702	0.2184	0.5376	1.3296
WT	0.0768	0.1472	0.2774	0.6906	1.3606
WT	0.0668	0.1412	0.2004	0.7546	1.4896
