# Supplementary material for: Caveolin-3 promotes glycometabolism, growth and proliferation in muscle cells
Source: PLoS One. 2017 Dec 5;12(12):e0189004. doi: 10.1371/journal.pone.0189004 (PMC5716543; doi:10.1371/journal.pone.0189004)
Supplement: S2 File — (PDF) [file pone.0189004.s002.pdf]

Glucose consumption(mmol/L) 48h

	NC	WT
	3.7	5.04
	2.29	5.04
	1.77	4.76
	2.09	3.72
	0.78	2.9
$\bar{x}$	2.126	4.292
s	1.054400	0.953370

Glycogen(μ g) 48h

	NC	WT
	7.53	9.01
	10.34	9.45
	12.26	7.24
	9.01	10.63
	6.65	14.33
$\bar{x}$	9.158	10.132
s	2.235130	2.643040

Glycogen(μ g) 6 days

	NC	WT
	4.2439	5.6491
	3.8768	4.9845
	2.9907	5.6491
	3.8768	4.3199
	4.3199	6.0922
$\bar{x}$	3.86162	5.33896
s	0.527999	0.693508

Glycogen(μ g) /10^6 cells

	NC	WT
	3.450336	3.766061
	3.178517	3.510206
	2.932054	3.103897
	3.285447	3.085638
	3.176392	3.807599
$\bar{x}$	3.2045492	3.4546802
s	0.188865	0.347791
