# Supplementary material for: Caveolin-3 promotes glycometabolism, growth and proliferation in muscle cells
Source: PLoS One. 2017 Dec 5;12(12):e0189004. doi: 10.1371/journal.pone.0189004 (PMC5716543; doi:10.1371/journal.pone.0189004)
Supplement: S3 File — (PDF) [file pone.0189004.s003.pdf]

	p-Akt/Akt		p-AMPK/AMPK		p-p70s6K/p70s6K	
	NC	WT	NC	WT	NC	WT
	0.5023	0.6958	0.3508	0.8155	0.0985	0.1233
	0.4112	0.6125	0.3801	0.4029	0.0762	0.0972
	0.3627	0.6028	0.4056	0.5421	0.0782	0.1175
			0.4613	0.8167	0.0657	0.0892
			0.46	0.7593	0.0711	0.0856
$\bar{x}$	0.4254	0.637033	0.4116	0.6673	0.0779	0.1026
s			0.0488	0.1859	0.0125	0.017

	GLUT4/GAPDH		p-GSK3β/GSK3β	
	NC	WT	NC	WT
	0.129057	0.156342	0.1092	0.1088
	0.1432	0.1488	0.3337	0.1404
	0.1129	0.212616	0.142	0.142
	0.051225	0.062326	0.1132	0.0761
	0.057974	0.063179	0.3128	0.2253
$\bar{x}$	0.098871	0.128652	0.2021	0.1385
s	0.04188	0.065017	0.1115	0.0554
